# Respiratory challenges and ventilatory management in different types of acute brain-injured patients

**DOI:** 10.1186/s13054-023-04532-4

**Published:** 2023-06-23

**Authors:** S. Frisvold, S. Coppola, S. Ehrmann, D. Chiumello, Claude Guérin

**Affiliations:** 1 Department of Anesthesia and Intensive Care, University Hospital of North Norway, Tromso, Norway; 2 Department of Clinical Medicine, UiT the Arctic University of Norway, Tromso, Norway; 3 Department of Anesthesia and Intensive Care, ASST Santi Paolo e Carlo, San Paolo University Hospital, Milan, Italy; 4 Department of Health Sciences, University of Milan, Milan, Italy; 5 Coordinated Research Center On Respiratory Failure, University of Milan, Milan, Italy; 6 CHRU Tours, Médecine Intensive Réanimation, CIC INSERM 1415, CRICS-TriggerSep F-CRIN Research Network, Tours, France; 7 INSERM, Centre d’étude Des Pathologies Respiratoires, U1100, Université de Tours, Tours, France; 8 Faculté de Médecine Lyon Est, Université Claude Bernard Lyon 1, 8 Avenue Rockefeller, 69008 Lyon, France

**Keywords:** Acute brain injury, Mechanical ventilation, Lung protective ventilation, Cerebral autoregulation, Neurogenic pulmonary edema, Acute respiratory distress syndrome

## Abstract

Acute brain injury (ABI) covers various clinical entities that may require invasive mechanical ventilation (MV) in the intensive care unit (ICU). The goal of MV, which is to protect the lung and the brain from further injury, may be difficult to achieve in the most severe forms of lung or brain injury. This narrative review aims to address the respiratory issues and ventilator management, specific to ABI patients in the ICU.

## Introduction

In patients with acute brain injury (ABI) the delivery of mechanical ventilation (MV) in the intensive care unit (ICU) involves adequate timing for intubation, lung protective ventilation (LPV), brain protection, and weaning. Unlike non-neurocritical patients, ABI patients usually have no primary respiratory indication for ventilator support, but often require prolonged MV, although they are often able to breathe spontaneously [1–3]. Better understanding the complex relationship between brain and respiration/ventilation and providing judicious respiratory support are critical issues.

## Respiratory challenges in patients with ABI

Severe ABI refers to a sudden event that results in brain damage and reduced perfusion leading to reduced alertness. ABI is heterogeneous and covers different subtypes, notably traumatic brain injury (TBI), subarachnoid hemorrhage (SAH), intracranial bleeding and hypoxic ischemic brain injury. Since the brain is surrounded by the inextensible skull, any change affecting brain volume would result in an increase in intracranial pressure (ICP) and impairment of cerebral blood flow upon depletion of the compensatory reserve. Therefore, perfusion is tightly regulated by cerebral autoregulation (CA) to preserve cerebral blood flow facing variations in systemic pressure and metabolism. CA is a potent modulator of cerebral vasoreactivity [4]. Changes in PaCO_2_ alters CA: both hypo and hypercapnia can induce cerebral ischemia from the reduction of perfusion through vasoconstriction or vasodilatation, respectively, the latter also promoting higher ICP.

Respiratory dysfunction is common in ABI and can result from either dysregulation of the breathing patterns or acute lung injury.

### Dysregulated respiratory centers function

The brainstem contains the respiratory centers responsible for regulating breathing. In ABI, the respiratory centers can be dysregulated either through direct damage to the brainstem or indirectly through an increase in ICP and mass effects due to cerebral hemorrhage or edema. Damage to the respiratory center leads to impaired respiratory drive [5].

They regulate the respiratory response to stabilize CO_2_, e.g., after an increase in PaCO_2_ and low pH in the cerebrospinal fluid or brain tissue [6]. The peripheral chemoreceptors located in the carotid body and the lungs affect drive by modifying the sensitivity and threshold of central chemoreceptors, providing faster and more intense response to changes in hypoxemia, PaCO_2_ and pH [7]. In addition, the lung mechanoreceptors are stretch receptors activated by lung inflation and inhibit central chemoreceptors that terminate inspiration during the Hering–Breuer inhibitory reflex [8]. The respiratory drive pathway can be compromised not only because of respiratory biochemical input (respiratory acidosis or hypoxemia) and/or a mechanical input such as atelectasis, but also from the primary brain injury [9].

### Acute lung injury

Apart from a damaged or dysregulated respiratory center, ABI is commonly associated with acute lung injury, such as neurogenic pulmonary edema (NPE), lung inflammation, acute respiratory distress syndrome (ARDS), aspiration pneumonia, ventilator-associated pneumonia (VAP) and lung contusion [10–12].

**Fig. 1:**
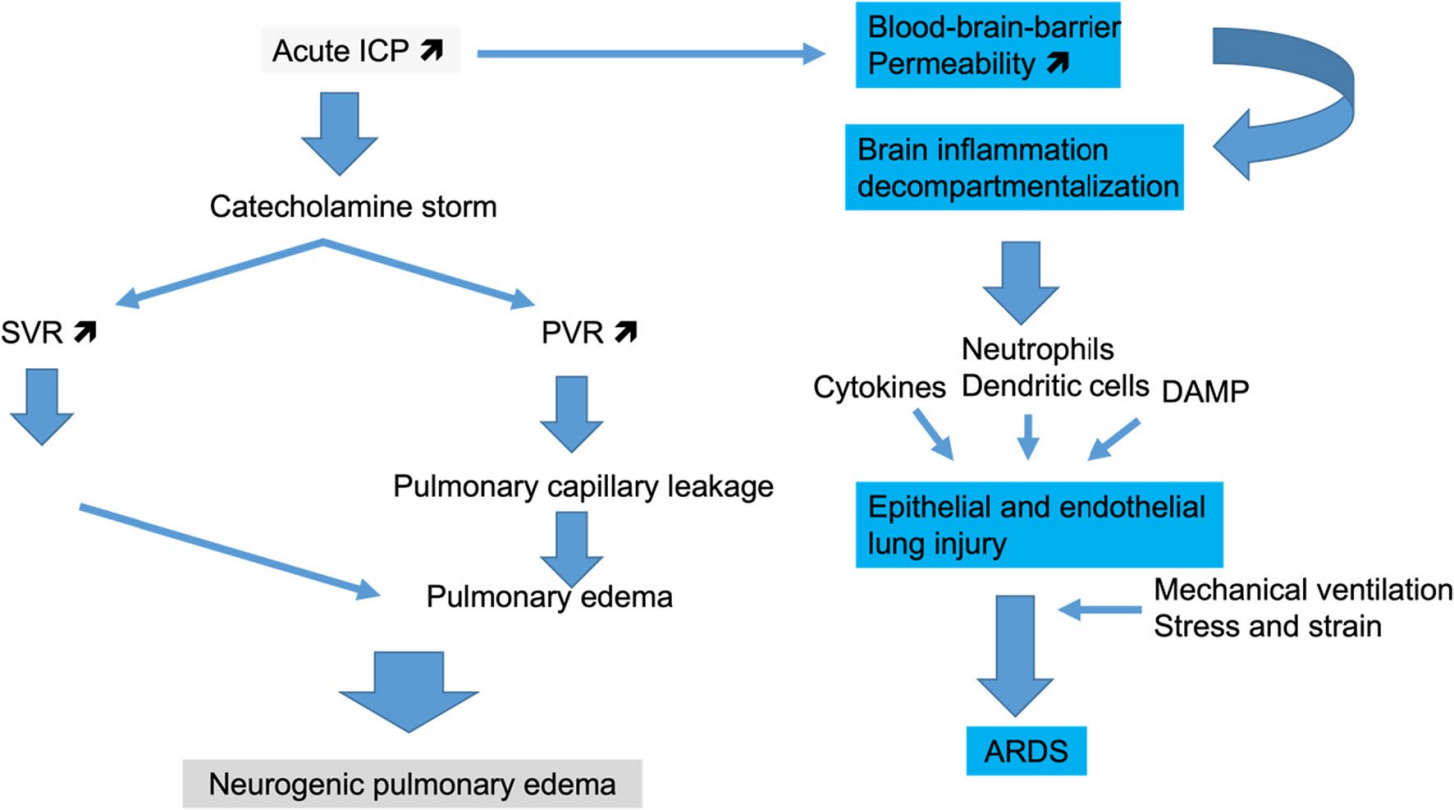
Pathways of acute lung injury directly related to acute brain injury. High intracranial pressure (ICP) can promote two different sequences of events that end up into neurogenic pulmonary edema or acute respiratory distress syndrome (ARDS). Both of them may coexist in a given patient. SVR: systemic vascular resistance, PVR: pulmonary vascular resistance, LV: left ventricle, DAMP: damage-associated molecules pattern

The most common ABIs associated with NPE are SAH, aneurysm rupture and TBI. NPE is typically characterized by the presence of respiratory distress, hypoxemia and bilateral alveolar opacities with diffuse infiltrates in both lungs in the absence of any other cause of respiratory failure [13, 14]. Thus, NPE is like the most severe form of acute hypoxemic respiratory failure,  e.g. the ARDS, but with a different pathophysiology (Fig. 1).

Typically, in the presence of increased ICP, a massive neural sympathetic discharge from anatomical regions such as the hypothalamus, the basal portion of the preoptic nucleus and periventricular system can occur [15]. This central sympathetic discharge likely induces pulmonary and systemic vasoconstriction or impairment in vascular permeability, promoting pulmonary edema [16].

In addition to the catecholamines storm that leads to NPE, a massive release of cytokines from the injured brain can contribute to cytokine-induced inflammation leading to ARDS [17–19]. The mechanisms of neuro-immunomodulation and ARDS have been recently reviewed [20]. Lung contusion due to multiple trauma that TBI patients frequently experience represents an additional risk factor for ARDS [21].

The reduced consciousness also makes the ABI patients more susceptible for aspiration pneumonia, impaired mucus clearance and VAP than ICU patients without ABI [19, 22, 23]. In a recent large European cohort of about thousand patients with TBI, one out of five developed VAP after a median interval of 5 days (interquartile range 3–7 days, indicating that 75% of episodes occurred in the first week of MV) [24]. Systematic performance of bronchoalveolar lavage within 24 h after intubation of trauma patients showed that 80% of samples grew some microorganisms and 30% are suspect of early pneumonia [22].

In summary, ABI can affect the respiratory system through different mechanisms and may interfere with evaluation of cerebral recovery and further delay the initiation of the weaning process. The respiratory support should simultaneously accomodate  the interplay between ICP, PaCO_2_ and cerebral perfusion, and deliver the lung protective ventilation, e.g. protecting the brain and the lung simultaneoulsy, which are potential contradictory goals.

## Ventilatory management of patients with ABI

**Fig. 2:**
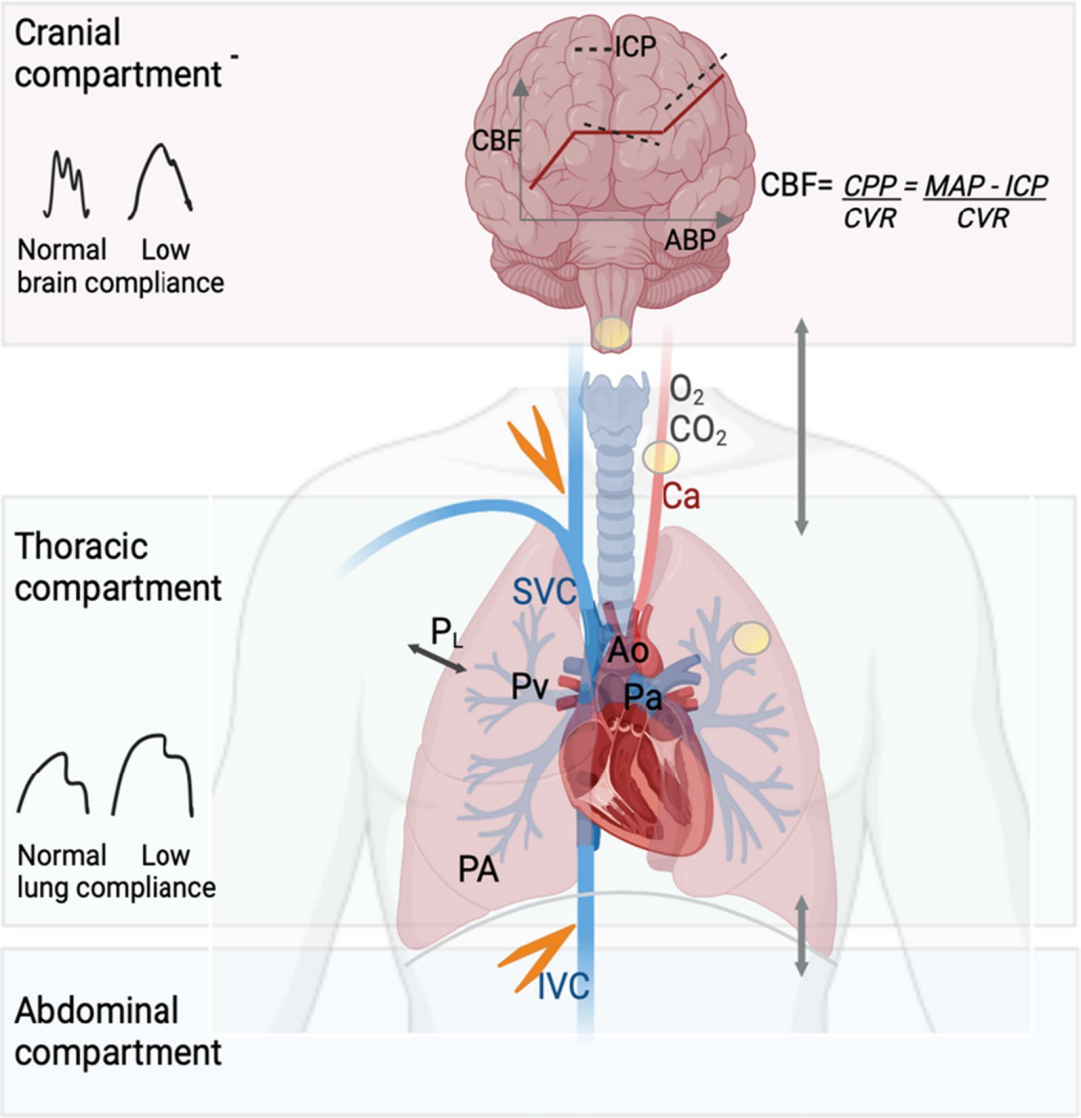
Schematic representation of the lung–brain interactions. During positive pressure mechanical ventilation, cerebral blood flow (CBF) can be reduced from different sources. The transmission of airway pressure to the cardiovascular structures depends on the pleural pressure and thus on the transpulmonary pressure (P_L_) and lung compliance. With normal lung compliance, the higher the airway pressure, the higher the right atrial pressure, which can lead to a reduction in venous return (orange flash). Increase in abdominal pressure counteracts this effect in normal conditions. Increased tidal volume increases pulmonary venous pressure (Pv). These changes result in lower right ventricular ejection volume, and thus, cardiac output (CO) will decrease. CO reduction is limited by the fact that the increased intrathoracic pressure will decrease the left ventricle afterload. Despite changes in CO and arterial pressure, cerebral autoregulation maintains CBF and intracranial pressure (ICP) within a certain range of arterial pressure. However, ICP is highly dependent on venous outflow from the cranial cavity. Positive pressure ventilation with increased right atrial pressure can reduce venous outflow from the cranial cavity and thereby increase ICP. In patients with impaired pulmonary compliance (i.e., severe acute respiratory distress syndrome), the effects of positive pressure mechanical ventilation on alveolar pressure (PA) and P_L_ are often attenuated. Hypoxemia (low PaO_2_) and hypercapnia (high PaCO_2_) both increase pulmonary artery pressure (Pa) and pulmonary vascular resistance, thereby increasing right ventricular afterload. Alterations in PaCO_2_, PaO_2_ and hydrogen ion also trigger chemoreceptors (yellow circles) to send signals to the respiratory center to regulate respiratory drive. At the level of cerebral circulation, hypercapnia increases CBF and hypocapnia has the opposite effect. The interaction between low brain compliance, cerebral autoregulation and different levels of CO_2_ has not been studied. Ao aorta, PaCO_2_ partial pressure of carbon dioxide, PaO_2_ partial pressure of oxygen, Ca carotid artery, cardiac output (CO), CBF cerebral blood flow, CPP cerebral perfusion pressure, CVR cerebral vascular resistance, ICP intracranial pressure, IVC inferior vena cava, MAP mean arterial pressure, Pa pulmonary artery pressure, PA alveolar pressure, Pv pulmonary venous pressure, PaO_2_ partial pressure of oxygen, P_L_ transpulmonary pressure, SVC superior vena cava

Figure 2 summarizes the brain–lung interactions during mechanical ventilation, the role of PaCO_2_ and the CA.

### Oxygen and carbon dioxide targets

The safety range of oxygenation targets in ABI patients is uncertain. Traditionally, the goal of oxygen supplementation in patients with ABI has been to avoid hypoxia [25]. Recent research supports the need to consider an upper limit of oxygen supplementation [26, 27]. The CENTER-TBI, which is a large European, multicenter observational study, showed that the median highest arterial oxygen partial pressure (PaO_2_) level during the first week after ICU admission was 134 mmHg [26]. The maximal and mean PaO_2_ were independently associated with an unfavorable functional neurologic outcome or death at 6 months. However, a cut-off for upper limit of PaO_2_ related to worse outcome was not defined [2]. By contrast, no difference in outcome based on PaO_2_ levels ranging from 60 to > 300 mmHg was found by others [28, 29]. A post hoc analysis of a randomized trial, identified PaO_2_ thresholds of 150 and 200 mmHg associated with better functional neurological outcome [30]. This is supported by a meta-analysis of observational studies on adult ABI patients where hyperoxemia (PaO_2_ cut-off point > 200 mmHg) was associated with poor neurological outcome [31]. Among randomized clinical trials (RCTs) performed in the ICU setting, the adverse effects of hyperoxemia has not been confirmed [32, 33]. Recent meta-analysis of RCTs did not support to set an upper safety PaO_2_ target in all-comer critically ill patients [34]. Cerebral oxygen supply by the use of microdialysis or brain tissue oxygen monitoring may serve as a marker for impending cerebral hypoxia [35]. Oxygen targeted by continuous monitoring of brain tissue oxygen will be addressed in an ongoing study [36]. To conclude, the role of excessive hyperoxemia is uncertain and avoiding it seems to be a reasonable strategy.

Hypocapnia is used to control acute bursts of high ICP. Recently, a retrospective analysis found that mild hypocapnia (30–34 mmHg) might be associated to better cerebrovascular reactivity and did not worsen cerebral energy metabolism [37]. Guidelines suggest that PaCO_2_ should be maintained in the normal or low normal reference range when ICP is high [3, 38]. The increased cerebral blood flow that follows hypercapnia might have a role in delayed cerebral ischemia (DCI), if ICP is controlled with external ventricular drainage [39]. Since patients with ABI are a heterogeneous group, future studies should identify those patients who may benefit from higher PaCO_2_ targets.

While invasive MV represents the standard management strategy to achieve above mentioned physiological goals for ABI patients, non-invasive methods could be an option in some circumstances.

### High-flow oxygen and non-invasive ventilation

There is limited evidence for use of high-flow oxygen and non-invasive ventilation (NIV) in ABI patients, at variance of acute hypoxemic respiratory failure in other patient populations [40, 41]. Coma is a contraindication for NIV unless it is due to acute hypercapnia in patients with chronic obstructive pulmonary disease [42]. Skull base fracture is a relative contradiction for use of high flow and NIV. Chest trauma might also complicate the use of NIV although some studies showed it feasible in case of isolated chest trauma [43]. To our knowledge, there are no published RCTs on the use of NIV in patients with TBI. Clinicians need to consider the individual patient´s clinical status and coexisting respiratory abnormalities when making decisions about the use of NIV. However, most often, ABI compromises the airway, warranting early intubation, sometimes after a brief attempt of NIV.

### Invasive mechanical ventilation

Indications for endotracheal intubation are to provide airway protection, treat hypoxemia and inadequate ventilation, management of brain edema with tight PaCO_2_ targets and reduction of cerebral metabolism.

Table 1 provides an overview of the ventilation management, fluid strategies, and indication for steroid use in patients with ABI with and without high ICP and/or ARDS based on the studies included in this review.

**Table 1:** Respiratory management of intubated ABI patients with and without intracranial hypertension and/or ARDS

Respiratory management (references)	Normal lung	Normal lung	ARDS	ARDS
	Normal ICP	High ICP	Normal ICP	High ICP
VT [44–46]	7–9 ml/kg PBW	7–9 ml/kg PBW	6–8 ml/kg PBW	6–8 ml/kg PBW
PEEP [47, 47–50]	5 cmH_2_O	5 cmH_2_O. If higher PEEP, surveillance of ICP/CPP/multimodal brain monitoring	At least 5 cmH_2_O, higher PEEP in more severe ARDS, titration based on plateau pressure, driving pressure, oxygenation and hemodynamic response to higher PEEP	At least 5 cmH_2_O, higher PEEP in more severe ARDS, titration based on plateau pressure, driving pressure, oxygenation and hemodynamic response to higher PEEP ICP/CPP/multimodal brain monitoring
PaO_2_ target (mmHg) [25, 26, 51]	Avoid hyperoxemia (PaO_2_ > 200)	PaO_2_ 80–200	PaO_2_ 80–120, depending of ARDS severity	PaO_2_ 80–200
PaCO_2_ target (mmHg) [38]	PaCO_2_ 35–45	PaCO_2_ 32–38	Permissive hypercapnia (< 60), depending on pH (> 7, 25)	Permissive hypercapnia contraindicated. Adjunctive therapy earlier ICP/CPP/multimodal brain monitoring
Prone position [52]	Not recommended	Not recommended	PaO_2_/FIO_2_ < 150 mmHg with PEEP ≥ 5 cmH_2_O	PaO_2_/FIO_2_ < 150 mmHg Case-by-case basis ICP/CPP/multimodal brain monitoring
Lung recruitment [53]	Systematic use, not recommended	Systematic use, not recommended	Systematic use, not recommended	Systematic use, not recommended
Fluid restriction strategy [54, 55]	Not recommended unless specific indication	Avoid positive fluid balance and target euvolemia for CPP management Avoid hypotonic fluids	Yes	Avoid positive fluid balance and target euvolemia for CPP management Avoid hypotonic fluids ICP/CPP/multimodal brain monitoring
Steroids [56, 57]	No indication except for specific indication	Higher mortality in TBI with high dose of methylprednisolone	Dexamethasone may be indicated	If TBI severity is the main problem steroids may be avoided. Steroids may be beneficial if ARDS is the dominant problem

### Specific types of ABI patients and ventilatory management

The management of ABI in the acute phase is mainly driven by the goal to ensure adequate cerebral perfusion, by cerebral perfusion pressure or ICP-oriented targets [38, 58].

The different forms of severe ABI have disease-specific characteristics that can influence ventilation management. Patients with severe TBI are most treated with ICP monitoring, which serves as a guide to ICU management [38, 59]. Strict low-range PaCO_2_ and PEEP targets are used for ICP control. Recently, CA as part of ICP management and the interaction of PEEP and LPV with CA has been evaluated [44, 47].

ICU management of patients with SAH targets prevention of rebleeding, intraventricular hemorrhage, and later-stage DCI [60]. In contrast to TBI, generalized contusion and cytotoxic edema are not the primary pathophysiological problem. Since these patients commonly have external ventricular drainage, strict PaCO_2_ targets for ICP monitoring are not required as often as in patients with TBI. In the DCI phase, microdialysis or brain tissue oxygen is sometimes used to detect local cerebral hypoxia and set PaO_2_ targets [61, 62]. An assessment of neurological function and reduced sedation is required to diagnose DCI. Spontaneous ventilation with the following broader PaCO_2_ target is therefore used more liberally than in TBI patients.

Most patients with severe intracerebral hemorrhage have systemic hypertension. Lowering blood pressure, rather than measures to lower ICP, has been the focus of general management in the ICU [63]. Treatment in the stroke unit is associated with a better outcome, but this does not necessarily improve when the patient is admitted to the ICU [64]. The indication for the use of ICP monitoring and targets is unclear and is often derived from the TBI literature.

### Ventilatory settings for ABI patients with no lung injury

In non-ARDS patients, no difference in patient outcome was found in two large multicenter RCTs between low (6 ml/kg predicted body weight) vs. intermediate (10 ml/kg) tidal volume (VT) [45] and low (5 cmH_2_O) vs. high (8 cmH_2_O) PEEP [48]. As these trials were not dedicated to ABI patients, a small proportion of the patients had ABI. Limiting VT with concomitant permissive hypercapnia is difficult to carry out with a simultaneous ICP control. Dead space reduction by replacing heat-moisture exchangers with heated-humidifiers is feasible and can set low VT without increasing PaCO_2_ [65]. High PEEP has shown contradictory results about the response related to brain and lung compliance [66–68]. Measurement of transpulmonary pressure could not clarify which ABI patients had adverse effect on high PEEP [44]. PEEP adjustment is therefore recommended only during rigorous ICP monitoring and curve analysis if brain compliance is suspected to be low [44]. The role of respiratory mechanics variables other than PEEP and VT has been explored in recent observational studies. A sub-analysis of the Target Temperature Management-2 trial found that respiratory rate, driving pressure, and mechanical power were independently associated with 6-month mortality in post-cardiac arrest survivors [69]. Mechanical power (MP) might also be associated with mortality in patients with ABI from other causes [70, 71]. A recent observational study found that the MP during the first week of MV was associated with poor outcome independently on oxygenation [71]. MP might also be related to PEEP-induced high ICP [44].

### Ventilatory management of ABI patients with concurrent ARDS

As detailed earlier in the ventilator management of patients with ABI some conflicting physiological goals may arise when aiming at protecting both the lung and the brain [72]. Typical, permissive hypercapnia as part of LPV may have cerebral side effects leading to a complex if not impossible evaluation of the benefit-risk ratio. While in patients without lung injury the value of strict LPV remains debated and cerebral physiology may predominantly drive patient management, the situation is more complex in case of established ARDS [11, 73]. Permissive hypercapnia might be feasible if ICP is controlled with external ventricular drainage. Another option is to increase respiratory rate up to the limit of Auto-PEEP or plateau pressure. Since respiratory rate contributes to MP, compensating for low VT by increasing respiratory rate might not be the solution for regulating PaCO_2_ in ABI. RCTs are required to assess the interplay between inflammatory and mechanical stress of the lungs in this population, as well as potential interventions and their impact on long-term outcomes on both cerebral and pulmonary recovery (weaning of MV).

Prone position in patients with moderate to severe ARDS is a cornerstone treatment that might be considered in patients with co-existing ABI. Since prone position might affect PaCO_2_ and venous return from the brain, ICP monitoring is strongly advised in acute, severe ABI [3, 74]. High ICP was a non-inclusion criterion in Proseva trial [52]. The role of alveolar recruitment maneuvers to improve oxygenation in ARDS is uncertain [53]. In patients with acute, severe ABI, the role of the recruitment maneuver with cardiopulmonary interaction, should lead to caution when using this maneuver.

Asynchronies between patient to ventilator might be the clinical consequences of the alterations in the respiratory drive or prolonged MV.

During assisted mechanical ventilation, the critical determinant of respiratory drive and work of breathing is the set peak flow. An insufficient peak flow is associated with higher drive and work of breathing. Low peak flow leads to air hunger and excessive peak flow leads to excessively short inspiratory time associated with asynchrony and breath stacking. Highest peak flows increase respiratory rates because of shortened inspiratory time. In fact, a shorter inspiratory time decreases the negative feedback derived from lung inflation, resulting in a higher ventilatory frequency. Physiologically, high volume or lung inflation reduces the respiratory drive and the peak flow becomes less relevant. Flow dyssynchrony is typical in situations of high drive or of activation of respiratory muscles after time-initiated ventilator cycles during controlled MV called “respiratory entrainment” secondary to a sustained activation of the Hering–Breuer reflex and C3, C5 spinal reflex leading to reverse triggering [77]. Luo et al. investigated the patient–ventilator asynchrony in mechanically ventilated brain-injured patients and found that the prevalence of asynchrony was 38% higher than in patients without brain injury [78], while the most prevalent type of asynchrony was ineffective triggering, characterized by a lower drive in terms of P0.1 values. The asynchrony index was similar after stroke, craniotomy for brain tumor or TBI, and significantly lower during pressure control/assisted ventilation than during other ventilation modes and higher during combined use of opioids and sedatives. Similarly, to non-neurological patients, asynchrony is a sign of uncoupling between the neuronal input and muscular efficiency and is associated with prolonged MV [78]. Recently, in patients with ABI, patient-ventilator asynchrony was monitored with esophageal pressure monitoring [78]. It was demonstrated that asynchrony, in particular ineffective trigging is common and associated with combination of analgesia and sedation strategy. The prevalence of ventilator-induced diaphragm dysfunction in brain-injured patients is likely to play an important role but remains to be investigated [79].

### Additional management

Two additional strategies relevant for the ventilatory management will be briefly discussed in this part: fluid balance and steroids.

*Fluid management in ABI*. The FACCT trial in ARDS patients (with no ABI) found that a restrictive strategy, as compared to the liberal fluid strategy, was similar in terms of patient mortality but was associated with less days spent under invasive MV [54]. In ABI patients, in addition to careful adjustment of the ventilator pressures, ensuring that patients are euvolemic might protect against the adverse effects of higher ventilator pressures and fluctuations in PaCO_2_. Fluid management must consider administrating fluids vs the risk of cerebral edema due to disruption of the blood brain barrier and cell damage.

In ABI patients with or without ARDS, the restrictive fluid strategy is therefore recommended to prevent any further brain edema. However, it is important to take care to avoid hypovolemia in order to achieve the cerebral perfusion pressure goal [55, 75].

*Steroids.* In patients with ARDS, steroids may play a beneficial role in the acute phase [56]. In TBI, the MRC-CRASH trial found that mortality was higher with methylprednisolone than with placebo; therefore, steroid use is not recommended [57]. In general, steroid use is not recommended in any form of ABI patients with acute cerebral swelling due to a lack of evidence [76].

### Weaning

Prolonged MV may further delay rehabilitation and ICU discharge, increasing the risk of long-term sequelae and complications with physical, psychological and psychiatric as well as cognitive symptoms as part of the post-intensive care syndrome which does also affect patients’ relatives [80]. Also, in patients with ABI, there has been increased focus on weaning practices and early rehabilitation [81, 82].

Uncertainty persists about the best approach to successful weaning of ABI patients and involves sedation practices, weaning criteria, and timing for tracheotomy. Traditionally, critically ill patients have been treated with deep sedation and immobilization, which prolong time to extubation [83]. This practice is in particularly common in patients with ABI, where deep sedation has been used to reduce cerebral metabolism, prevent intracranial hypertension and for fever control. Recently, ABCDEF bundle (Assess, prevent, and manage pain; Both spontaneous awakening and breathing trials: Choice of Analgesia and Sedation; Delirium assess, prevent, and manage; Early Mobility and Exercise; Family engagement/ empowerment) received acknowledgement within intensive care medicine [84]. Among ABI patients, data are lacking to precisely decipher the relative contributions of sequelae of the initial cerebral insult and intensive care acquired long-term neurological, muscular and cognitive symptoms. Patients with ABI have higher extubation failure than patients without [85, 86].

Several factors may contribute to extend the duration of MV, some are non-modifiable such as the brain injury itself, recovery of consciousness being one key factor for weaning readiness; others, like management-related factors may be modified such as deep and prolonged sedation and screening practice for weaning readiness [87, 88]. Once extubated, patients with ABI are at high risk of re-intubation, mainly because of respiratory and airway failure due to dysphagia, lack of muscle strength and poor cough. In a large international observational study, that evaluated 1512 patients with ABI who were ventilated more than 24 h with an initial Glasgow coma scale (GCS) ≤ 12, 19% of patients were reintubated within 5 days of extubation. Authors identified predictors of extubation failure at day 5 which were combined in a score which may be easy to use at the bedside and comprising the following variables associated with extubation success: TBI, vigorous cough, gag reflex, swallowing attempts, endotracheal suctioning less than twice per hour, GCS motor component at 6, body temperature normal or low. The score area under the receiver operating characteristics curve was 0.65 (95% confidence interval, 0.53–0.76) in the validation cohort, clinical evaluation of cut-offs with high or low positive predictive value may be warranted to decipher potential clinical use of this score. Of note, 21% of the patients of this large international cohort did not undergo usual weaning toward extubation but underwent direct tracheostomy. The optimal timing of eventual tracheostomy of ICU patients has been the matter of long-lasting debates, with a current lack of firm benefit of early tracheostomy. A recent large scale retrospective study including 1538 patients, specifically evaluated timing of tracheostomy among the subgroup of 498 patients with a GCS below 8 at ICU admission and showed a lack of significant link between tracheostomy timing and patient outcome [89]. An observational study on 1358 patients with TBI showed an association between late tracheostomy and poor neurological outcome [90]. However, this finding was not confirmed by the SETPOINT2 RCT on patients with stroke [91]. Current guidelines recommend considering tracheostomy in patients who failed an extubation or have persistent reduced level of consciousness with no recommendation on optimal timing of tracheostomy [3]. Focusing on measures to reduce sedation/analgesia with early weaning of patients with severe ABI are the target of future studies (NCT04291235, NCT04080440).

## Conclusions

Neurologically ill patients have specific challenging respiratory problems and lung–brain interactions. Optimal delivery of MV has not been studied extensively in this setting and RCTs are scarce. Recent interest in the combination of neuro- and respiratory monitoring and large multicenter trials in ABI patients advances our knowledge in terms of optimized treatment and outcomes. The future will offer the opportunity to combine physiological studies and the use of big data analysis to identify predictors and optimize ventilation strategies for patients with severe ABI.

## Data Availability

Data sharing is not applicable to this article as no datasets were generated or analyzed during the current study.

## Abbreviations

ABI: Acute brain injury
ARDS: Acute respiratory distress syndrome
CA: Cerebral autoregulation
DCI: Delayed cerebral ischemia
GCS: Glasgow coma scale
ICP: Intracranial pressure
ICU: Intensive care unit
LPV: Lung protective ventilation
MP: Mechanical power
MV: Mechanical ventilation
NPE: Neurogenic pulmonary edema
NIV: Non-invasive ventilation
PaCO_2_: Arterial carbon dioxide partial pressure
PaO_2_: Arterial oxygen partial pressure
PEEP: Positive end-expiratory pressure
RCT: Randomized controlled trial
SAH: Subarachnoid hemorrhage
TBI: Traumatic brain injury
VT: Tidal volume
VAP: Ventilator associated pneumonia
